# Distribution of Al, Fe, Si, and DOC between size fractions mobilised from topsoil horizons with progressing degree of podzolisation

**DOI:** 10.1038/s41598-022-12616-4

**Published:** 2022-05-19

**Authors:** Agnes Krettek, Mathias Stein, Thilo Rennert

**Affiliations:** Department of Soil Chemistry and Pedology, Institute of Soil Science and Land Evaluation, University of Hohenheim, 70593 Stuttgart, Germany

**Keywords:** Biogeochemistry, Carbon cycle, Element cycles, Environmental sciences

## Abstract

Aluminium, Fe, Si, and dissolved organic C (DOC) accumulate in the subsoil of Podzols after mobilisation in the topsoil. We conducted laboratory experiments with topsoil horizons with progressing degree of podzolisation by irrigation with artificial rainwater at varying intensity and permanence. We monitored the concentrations and distribution of mobilised Al, Fe, Si, and DOC between size fractions (< 1000 Dalton, 1 kDa– < 0.45 µm, and > 0.45 µm). Total eluate concentrations were increased at the onset of the experiments and after the first irrigation interruption, indicating non-equilibrium release. There was no statistical effect of the degree of podzolisation on element concentrations. Release of Al, Fe, and DOC was mostly dominant in the fraction 1 kDa– < 0.45 µm, indicating metals complexed by larger organic molecules and colloids. Silicon released was dominantly monomeric silicic acid < 1 kDa. Particularly with the least podzolised soils, Al and Si concentrations < 1 kDa might have been controlled by short-range ordered aluminosilicates, while their transport in colloidal form was unlikely. Our study pointed to both quantitative and qualitative seasonality of element release during podzolisation, to decoupling of Al and Si release regarding size, and to different minerals that control element release as a function of the degree of podzolisation.

## Introduction

Podzols, according to the World Reference Base for Soil Resources^1^, and Spodosols^2^ are soils accumulating soil organic matter (SOM) and aluminium (Al) or iron (Fe) species or both in the subsoil. Accumulation of these soil constituents is the result of their previous mobilisation in organic and mineral topsoil horizons. Permeable substrate, sufficient seepage water, acidic parent material, vegetation providing nutrient-poor litter, and low soil temperature promote mobilisation^3^. Mobilisation comprises: (i) release of Al and Fe ions by weathering of silicates and metal oxides at low pH and subsequent transport of the ions complexed with dissolved organic matter (DOM); (ii) formation and transport of colloidal short-range ordered aluminosilicate (SROAS) sols (e.g. proto-imogolite); (iii) reduction of Fe(III) and transport as a complex with DOM; (iv) formation and transport of DOM and organic colloids^3–5^.

Translocation of SOM, Al, Fe, and silicon (Si) species in Podzols with the soil solution as transport medium is beyond dispute, while the physical state and the analytical allocation of the transported elements differs. The recent operational definition of dissolved species bases upon filtration at 0.45 µm^6^. However, Fe-oxide particles, partially associated with SOM, amorphous silica, SROAS, and SOM particles < 0.45 µm in size have been detected in the soil solution^7–9^. Vice versa, transport of particles > 0.5 µm, including Al, Fe, Si, and SOM, in the soil solution of different Podzol horizons has been reported, together with the dissolved fraction^10^. Furthermore, both the amount of transported species and their physical state (dissolved or colloidal in varying sizes) in Podzol soil solutions depended on the amount of percolating water that increases for instance after snow melt or during heavy rain events^8,11^. Percolation that was more intensive or started after a period of draught or frost promoted particularly vertical colloidal/particulate transport in Podzols^8,12^. This effect is caused by release of precipitated colloids, hydrodynamic forces caused by infiltration of the seepage water, shrinking and swelling in wet-dry cycles, mechanical strain during freezing, and onset of preferential flow^8,13^. Percolation intensity, duration, and frequency does not only affect the release of colloidal/particulate matter, since mobilisation of adsorbed species or species from mineral dissolution, resulting in dissolved species, are frequently rate-limited processes^8,11,12^.

Particularly dissolved Al and Si species are multifaceted. Complexation of Al by DOM involves low-molecular-weight organic compounds (LMWOC), for instance particular carboxylic and aromatic acids, which are known to percolate from the litter layers into Podzol mineral horizons^14^, and larger organic molecules, interpreted as humic and fulvic acids, the molecular mass of which exceeds 1000 Dalton (1 kDa)^5^. Actually, Al-DOM complexes passing a 1 kDa dialysis membrane are considered ‘truly dissolved’, while metal-DOM complexes > 1 kDa are considered colloidal^15^. Similarly, the molecular-mass cut-off 1 kDa (approximately 1 nm in size) corresponds to the size discussed for monomeric silicic acid^16,17^, enabling the differentiation of dissolved monomeric from colloidal oligomeric/polymeric silicic acid. Consequently, information on the physical state, that is, truly dissolved or colloids of varying size, and on speciation of elements in the soil solution is a valuable tool to study recent processes of podzolisation^17–19^, particularly those in which colloidal transport of SROAS-like sols may be involved^20,21^.

Apart from the variety of species transported by the soil solution, and affected by intensity and permanence of water flow, the question arises whether the progress of podzolisation affects the speciation of elements in the soil solution. That means, does the distribution of elements among physical states (dissolved, colloidal) in the solution of mineral topsoil horizons vary, depending on the degree of podzolisation? Furthermore, does the distribution of element speciation provide information on the type of transport and the potential contribution of SROAS? Answering these research questions was the aim of this study. We took advantage of five soil profiles, developed from identical parent material, with progressing degree of podzolisation. We studied the effects of varying intensity and permanence of irrigation on the release of Al, Fe, Si, and DOM from mineral topsoil horizons by fractionating and analysing the eluates by size; < 1 kDa, 1 kDa– < 0.45 µm, and > 0.45 µm.

## Materials and methods

### Site, sampling, and soils

We conducted our study with L, O, and A horizons taken collectively from five soil profiles located within a radius of < 500 m on the Lower Rhine Plain in NW Germany (51°10′16″N, 6°11′40″E), developed from aeolian sand over Pleistocene sediments of the main terrace of the river Rhine. The soils represent a sequence of increasing morphological podzolisation, from a Dystric Arenosol to an Albic Podzol (Table 1)^22^. The ascending designations ‘P1’ to ‘P5’ (Table 1 and in the article) follow the increasing degree of podzolisation. We took samples in duplicate by vertically driving PVC cylinders (inner diameter 10 cm, length 25 cm) into soil, until the lower boundary of the AE or EA horizon was reached. The cylinders contained the mineral A horizons of varying depth (Table 1), together with the overlying organic L and O horizons.

**Table 1:** Selected properties of soils^22^ used in irrigation experiments (pv, pore volume; SOC, soil organic carbon). The subscripts index element extraction by dithionite-citrate-bicarbonate (DCB), oxalate (Ox), and citrate (C). Two pore volumes are given for Sample P4 because of varying thickness of the mineral horizon between the two replicates.

Soil type	Mineral horizon	Thickness (cm)	pv (mL)	pH (CaCl_2_)	Sand (g kg^−1^)	Silt (g kg^−1^)	Clay (g kg^−1^)	SOC (g kg^−1^)	Fe_DCB_ (mg kg^−1^)	Fe_Ox_ (mg kg^−1^)	Fe_C_ (mg kg^−1^)	Al_Ox_ (mg kg^−1^)	Al_C_ (mg kg^−1^)	Si_Ox_ (mg kg^−1^)
Dystric Arenosol; P1	AE	8	420	2.7	980	18.4	1.6	93.9	4725	2150	2165	940	945	23
Dystric Brunic Arenosol; P2	AE	8	419	2.7	996	3.5	0.5	52.9	3817	820	850	273	310	13
Dystric Brunic Arenosol; P3	AE	8	429	3.0	997	2.7	0.3	27.3	3815	983	1075	260	290	< 3
Dystric Brunic Arenosol; P4	AE	12 10	643 536	2.9	997	2.5	0.5	24.9	3845	1155	1015	340	330	3
Albic Podzol (Arenic); P5	EA	10	519	2.9	995	2.0	3.0	17.8	219	188	210	140	145	< 3

All soils were very sandy, strongly acidic, and with decreasing SOC contents in the mineral topsoil horizon with progressing podzolisation (Table 1). The contents of oxalate-extractable Si, potentially indicating SROAS, were low in all samples. Notably, the contents of oxalate-extractable and citrate-extractable Al and Fe were almost identical, indicating organic and mineral sources of the released metals^22,23^. Given the very sandy texture, quartz was the dominant mineral, with accessory feldspars and clay minerals (illite, chlorite, interstratified minerals and kaolinite)^22^. 

### Irrigation experiments and analyses

**Figure 1:**
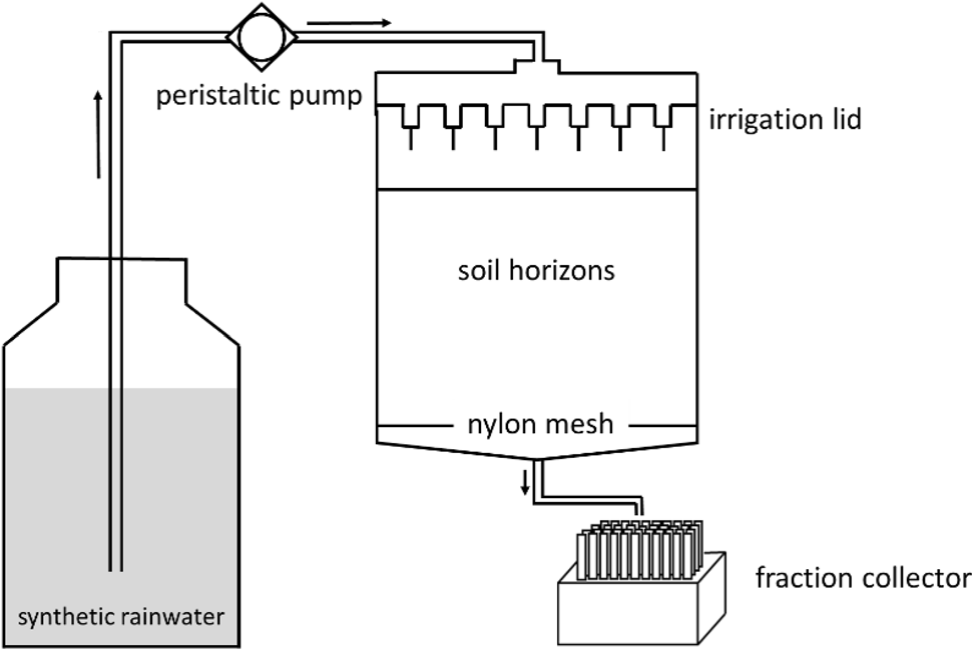
Schematic illustration of the experimental setup of the irrigation experiments.

We covered the bottom of each PVC cylinder with a nylon mesh and placed the cylinders in outlet funnels (Fig. 1). A peristaltic pump (Ismatec MCP, Cole-Parmer, Wertheim, Germany) supplied artificial rainwater (pH 5.3; 0.07 mM Ca(NO_3_)_2_, 0.04 mM NaNO_3_, 0.12 mM (NH_4_)_2_SO_4_)^24^ to the irrigation unit. We ran the irrigation experiments with free drainage at two varying pore water velocities (q = 1 mm h^−1^ (slow) and q = 10 mm h^−1^ (fast)), to simulate phases of less percolation (e.g. during steady rain) and intensive percolation (e.g. during heavy rain or snowmelt). To enforce physical and chemical non-equilibrium release of soil constituents, we interrupted the flow after irrigation for two or 20 days. The experiments consisted of the consecutive steps fast irrigation—flow interruption for 20 days—slow irrigation—flow interruption for two days—fast irrigation. The duration of the irrigation phases varied because of different thicknesses of the mineral horizons (Table 1), that is, the exchange of a pore volume lasted different times among the soils. We approximated the pore volume (pv) using the soil bulk density ρ_bulk_, determined with separate soil cylinders. As all soil samples almost completely consisted of sand (Table 1), we approximated the soil density ρ_soil_, using the density of quartz (ρ_qz_ = 2.65 g cm^−3^) and the SOM contents (SOM = 2 × SOC (Table 1); ρ_SOM_ = 1.4 g cm^−3^). Thus, the porosity ε was (ε = 100 × ρ_bulk_/ρ_soil_), and pv = ε × V_soil_ (V_soil_ resulted from the area of the soil cylinder and the thickness of horizons within the individual cylinders).

In each irrigation phase, we took samples from 10 pv, the first after 0.5 pv, the last after 10 pv eluted. The sampling interval was 0.5 pv so that 60 eluate samples were collected for each soil cylinder, using fraction collectors. The samples used for analysis represented the last 150 mL of a pore volume (of 0.5 pv for the first sample).

We divided every second eluate sample (n = 30 for each irrigation experiment) into three fractions that were individually analysed. For fractionation, we transferred the eluate into a 250 mL-polypropylene beaker, in which we placed a dialysis membrane (pre-treated cellulose, molecular mass cut-off 1 kDa; Spectra Por 7 (Repligen, Waltham, USA)) filled with 10 mL of ultrapure water for 24 h. Equilibration with 1 kDa membranes is achieved after 12 h^25^. The fraction < 1 kDa represents truly dissolved Al and Fe ions, or complexed with LMWOC, and monomeric silicic acid as pointed out before.

An aliquot of the remaining 150 mL of eluate samples (> 1 kDa; retentate) was filtered with single-use syringe filters (cellulose acetate, 0.45 µm filter size). The filtrate comprised the fraction < 1 kDa, dissolved species, metals complexed by larger/colloidal organic molecules, and organic and inorganic colloids < 0.45 µm^15^. The unfiltered retentate comprised the entire fraction < 0.45 µm, together with particles > 0.45 µm. All fractions and the total eluate samples were analysed individually for their organic and inorganic composition. The pH of the total samples was measured potentiometrically. Total eluate samples and the unfiltered retentate were acidified with 5 M HCl. Inductively coupled plasma optical emission spectrometry (ICP-OES; 5110 SVDV, Agilent, Santa Clara, USA) or mass spectrometry (ICP-MS; NexION 300X, Perkin Elmer, Waltham, USA) were used to quantify Al, Fe, and Si in the eluates. We used ICP-OES when concentrations exceeded 500 µg L^−1^ (Al or Si), or 50 µg L^−1^ (Fe). Less concentrated eluates were analysed by ICP-MS, with detection limits of 1 (Al, Fe) and 10 µg L^−1^ (Si), respectively. Eluate DOC concentrations were determined by catalytic high-temperature oxidation using a DIMATOC 2100 (Dimatec, Essen, Germany). The concentrations and size distributions of elements presented in the following refer exclusively to a certain fraction. For instance, the retentate after 0.45 µm filtration included originally species < 1 kDa. As we analysed the fraction < 1 kDa separately, we could correct the filtrate < 0.45 µm for the fraction < 1 kDa so that we could calculate species concentrations of the fraction 1 kDa– < 0.45 µm. The same applied for the fraction > 0.45 µm.

### Data evaluation

We used IBM SPSS Statistics 27 for statistical analyses. Two-way analysis of variance was conducted to determine the effects of progressive Podzol development (P1 to P5) and cylinder replicate on the elemental concentrations among eluates and the distribution of elements among size fractions (p < 0.05). We used Levene’s test to assess homogeneity of variances. Depending on the homogeneity of variances, we used the Tukey (p > 0.05) or Games-Howell (p < 0.05) test to check t-test variations in post-hoc analyses.

We evaluated the data of the eluate fraction < 1 kDa, considering the composition of the synthetic rain water, eluate pH, and concentrations of DOC, Al, and Si to determine the activity of Al^3+^ ions and silicic acid, using Visual MINTEQ 3.1^26^. Activities were corrected according to the Davies equation. As the organic species < 1 kDa were LMWOCs, we did not apply approaches provided with Visual MINTEQ that describe DOM as fulvic and humic acids (e.g. the Stockholm Humic Model), since these species comprise compounds of larger size/molecular mass. Instead, we converted molar DOC concentrations to those of citric or acetic acid, two LMWOCs, which are commonly present in the soil solution percolating into Podzol A horizons, and dominating the DOM fraction of carboxylic acids^14,27,28^. We considered these LMWOCs as models of DOM < 1 kDa present in Podzol soil solutions, not only because of their quantitative importance, but also because citric acid was found the most important complex former when comparing analytical and modelling data^29^. Even in early states of podzolisation, the easily soluble DOM fraction represents OM transported into the subsoil and SOM detected in illuvial B horizons of developed Podzols^30^.

## Results and discussion

### Total and relative release of elements (Al, DOC, Fe, Si) from soil during irrigation

**Figure 2:**
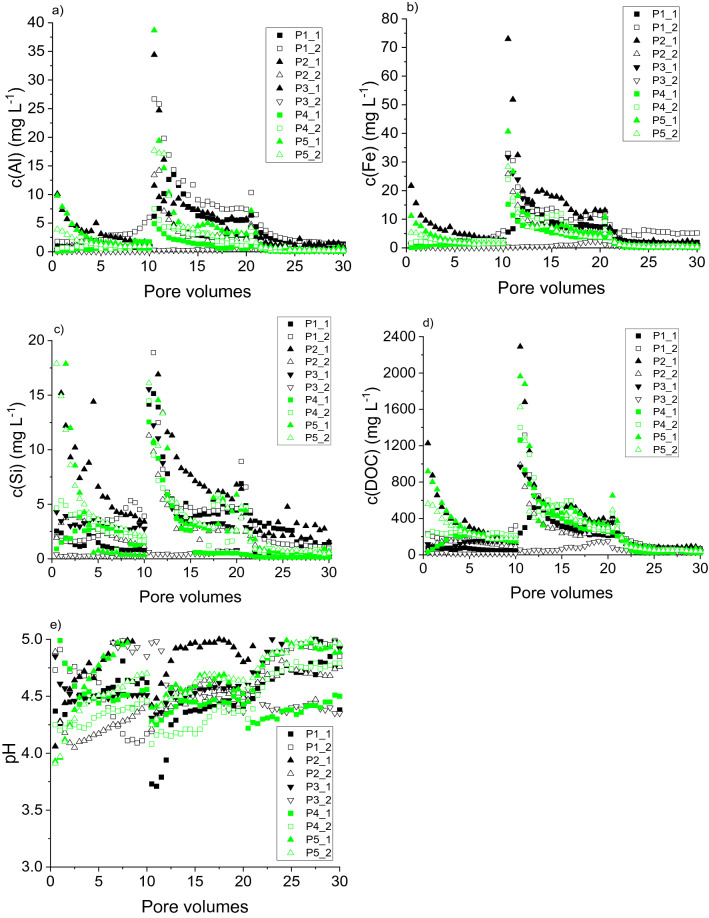
Elemental concentrations (**a–d**) and pH in eluates from irrigation experiments with soils with increasing degrees of podzolisation (P1 to P5). ‘_1’ and ‘_2’ denote duplicate experiments. (**a**) Aluminium, (**b**) Iron, (**c**) Silicon, (**d**) Dissolved organic carbon, (**e**) pH.

Total eluate concentrations of all elements under study responded to changes in the irrigation regime (Fig. 2). We are aware that pore volumes given in the figures are an approximation, but this kind of presentation makes an optical comparison of the different experiments more comprehensive. Apart from experiment P3_2 (Fig. 2), that is, one of the duplicates of the irrigation experiment with the horizons of soil profile P3 (Table 1), release of all elements revealed a distinct first flush at the very beginning of irrigation, with increased concentrations that decreased in the course of irrigation. The concentrations of all elements in the experiments with P2 differed significantly between the duplicates, and similar with P3 (no significant difference for Al). Furthermore, there was no significant effect of the degree of podzolisation on the total element concentrations.

First flush is a common feature of element release in the initial phase of column experiments, pointing to mobilisation of particles or rate-limited desorption or dissolution^12,31,32^. Non-equilibrium release became more evident after the longer interruption of irrigation after 10 pv eluted, and to smaller extent after the shorter irrigation after 20 pv eluted (Fig. 2). Preferential flow responsible for the concentration patterns should be of minor importance in the extremely sandy soils, as previously discussed^12^. Concentrations after the first interruption exceeded those of the first flush and were partially very high, for instance c(Fe) > 70 mg L^−1^ (Fig. 2b) and c(DOC) > 2300 mg L^−1^ (Fig. 2d). Eluate pH (Fig. 2e) was generally higher than soil pH (Table 1), possibly because of irrigation with the less acidic synthetic rainwater. However, the pH of the eluates varied between the soils roughly from 4 to 5. The partial decrease in pH after irrigation interruptions excludes reducing conditions during the interruptions owing to the reciprocal relationship of pH and the redox potential. Instead, decreasing pH indicates the release of acidifying substances, particularly Al^3+^ ions. Differences in pH between the soils may point to variable processes inducing the release of species in different physical states. However, these variations were not systematic and a function of the degree of podzolisation.

Based on total-eluate data, it is not possible to differentiate between physical non-equilibrium processes, inducing the release of colloids of varying size, and chemical non-equilibrium, inducing the release of dissolved species. As this differentiation would possibly enable mechanistic conclusions on podzolisation processes, we fractionated the eluates by size prior to chemical analyses.

Based on ANOVA, there was no effect of the degree of podzolisation on the concentration patterns of any element in any size fraction (< 1 kDa, 1 kDa– < 0.45 µm, > 0.45 µm), neither was there a difference between any duplicate regarding Fe and DOC concentrations. The same applied to most Al and Si eluate concentrations, with the exceptions of P1 (Al, 1 kDa– < 0.45 µm, > 0.45 µm) and P2 (Si, > 0.45 µm), where the concentrations differed significantly between the duplicates. The lack of significant differences between eluate concentrations in experiments with soils of varying degrees of podzolisation may be the result of very similar pH conditions and patterns in all experiments (Fig. 2e), and the similarly mostly low concentration levels throughout the irrigation experiments. On the other hand, the contents of oxalate-extractable Al as well as dithionite-extractable and oxalate-extractable Fe, which may be considered as potential sources of dissolved ions in the eluates, largely differed between the soils (Table 1). However, eluate concentrations did not reflect these differences significantly.

Effects of the irrigation regime on eluate concentrations were obvious in all fractions of all elements, albeit to varying extent (Supplementary Figs. S1–S4). Concentrations of Al were particularly increased after the first flow interruption with the 1 kDa– < 0.45 µm fraction (Supplementary Fig. S1b), likely contributing to the decrease in pH observed (Fig. 2e). Rate-limited release of ‘truly dissolved’ Fe (< 1 kDa) was restricted to P1_2 (Supplementary Fig. S2a). First-flush effects and increased concentrations after the first irrigation interruption were clear for all Si fractions (Supplementary Fig. S3), indicating release of monomeric, polymeric silicic acid, and Si-containing colloids/particles. The pronounced first flush observed for total eluate Si (Fig. 2c) was caused by ‘truly dissolved’ Si < 1 kDa (Supplementary Fig. S3a). Generally, desorption of monomeric silicic acid (Si < 1 kDa) is favoured to that of polymeric silicic acid, as it has a lower binding affinity for mineral surfaces so that Si is predominantly transported as monomeric silicic acid^33^. Increased concentrations of ‘truly dissolved’ Si after the first irrigation interruption may indicate mineral dissolution and desorption of monomeric silicic acid that is favoured at decreasing pH^33^ (Fig. 2e). Additionally, polymeric silicic acid adsorbed on mineral surfaces is stabilised at pH < 6, but simultaneously releases monomeric silicic acid into the soil solution^34^. Dissolution of amorphous silica in gel-like coatings on minerals formed at pH < 3.6^35^ may contribute to Si in the 1 kDa– < 0.45 µm fraction under experimental pH conditions > 4. The partially strongly increased total-eluate DOC concentrations (Fig. 2d) were not solely caused by organic particles, as the peak DOC concentrations in the fraction 1 kDa– < 0.45 µm exceeded those of the fraction > 0.45 µm (Supplementary Figs. S4b, c).

Correlations between metal and DOC concentrations were particularly close for the fractions < 1 kDa and 1 kDa– < 0.45 µm (Supplementary Fig. S5). The correlations are consistent with the vast majority of Al (< 0.2 µm) in Podzol soil solutions present in organic complexes^36^. Consistently, a major part of Al and Fe in the fraction > 3 kDa in Podzol O- and E-horizon soil extracts from Podzols was associated with DOM^37^. For the fraction > 0.45 µm, these correlations for both metals became closer with increasing degree of podzolisation. However, it remains unclear, whether the correlations actually reflect interactions between Al and Fe ions with organic particles, or whether they reflect the temporal coincidence of the release of mineral metal-containing particles and organic particles. Metal and DOC concentrations determined in lysimeter solutions (< 0.45 µm) from Podzols peaked together during a two-year monitoring^37^. Compiled DOC:metal ratios necessary for precipitation of solid phases^5^ fall approximately below 10, which is smaller than the ratios detected in any eluate < 1 kDa (> 33) and 1 kDa– < 0.45 µm (> 83) in our study, clearly showing metal mobilisation in our experiments. Our results confirm previous findings^37^ on both the values of DOC:metal ratios in the fraction 3 kDa– < 0.45 µm in Podzol soil solutions and the sequence of the ratios, Fe:DOC > Al:DOC.

**Figure 3:**
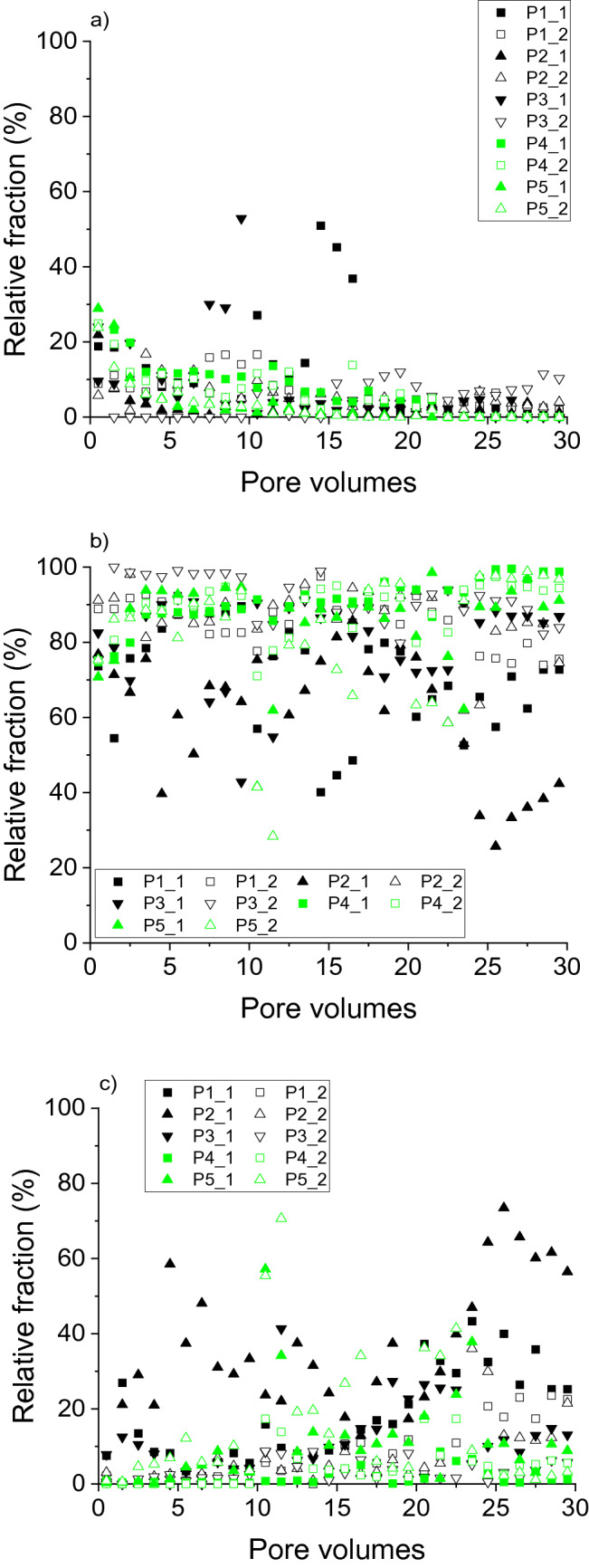
Relative distribution of aluminium eluted from soils with increasing degrees of podzolisation (P1 to P5) in irrigation experiments between fractions. (**a**) < 1 kDa, (**b**) 1 kDa–0.45 µm, (**c**) > 0.45 µm. ‘_1’ and ‘_2’ denote duplicate experiments.

**Figure 4:**
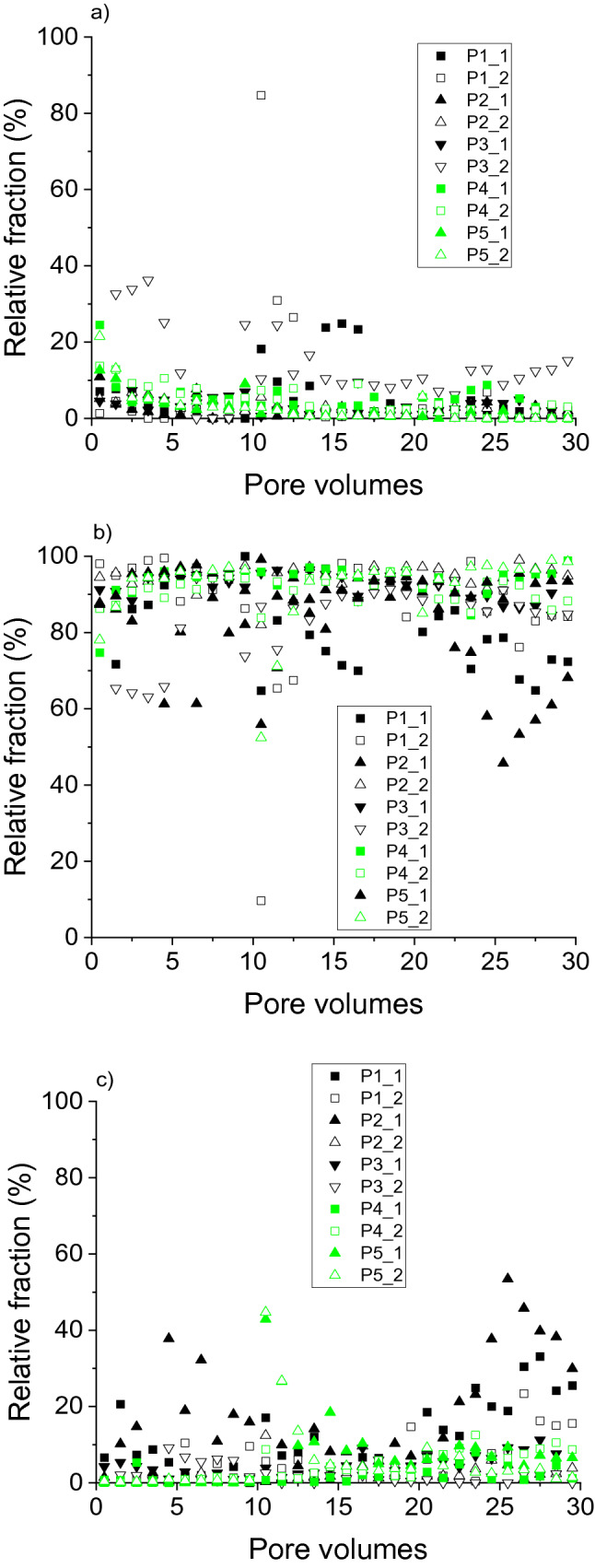
Relative distribution of iron eluted from soils with increasing degrees of podzolisation (P1 to P5) in irrigation experiments between fractions. (**a**) < 1 kDa, (**b**) 1 kDa–0.45 µm, (**c**) > 0.45 µm. ‘_1’ and ‘_2’ denote duplicate experiments.

Apart from the absolute element concentrations in the eluate fractions, the relative distribution of elements between the fractions provided information on potential differences in the dynamics of the elements. For mostly the entire duration of the irrigation experiments, the proportion of Al in the fraction < 1 kDa did not exceed 20% (Fig. 3a). Particularly with P4 and P5, the 1 kDa– < 0.45 µm fraction dominated Al distribution (Fig. 3b), similar to results for soil water extracted from Podzol E horizons^37^. However, the contribution of particulate Al (> 0.45 µm) to total Al export from the soils under study increased towards the end of the experiments, that is, after changes in irrigation intensity and permanence. The distribution of Fe between the size fractions during the irrigation experiments (Fig. 4) was similar to that found for Al (Fig. 3). Thus, the relative distribution of both metals coincided with complexation with larger organic molecules, which was expressed in both the size distribution, and the correlation with DOC concentrations 1 kDa– < 0.45 µm, as described before.

**Figure 5:**
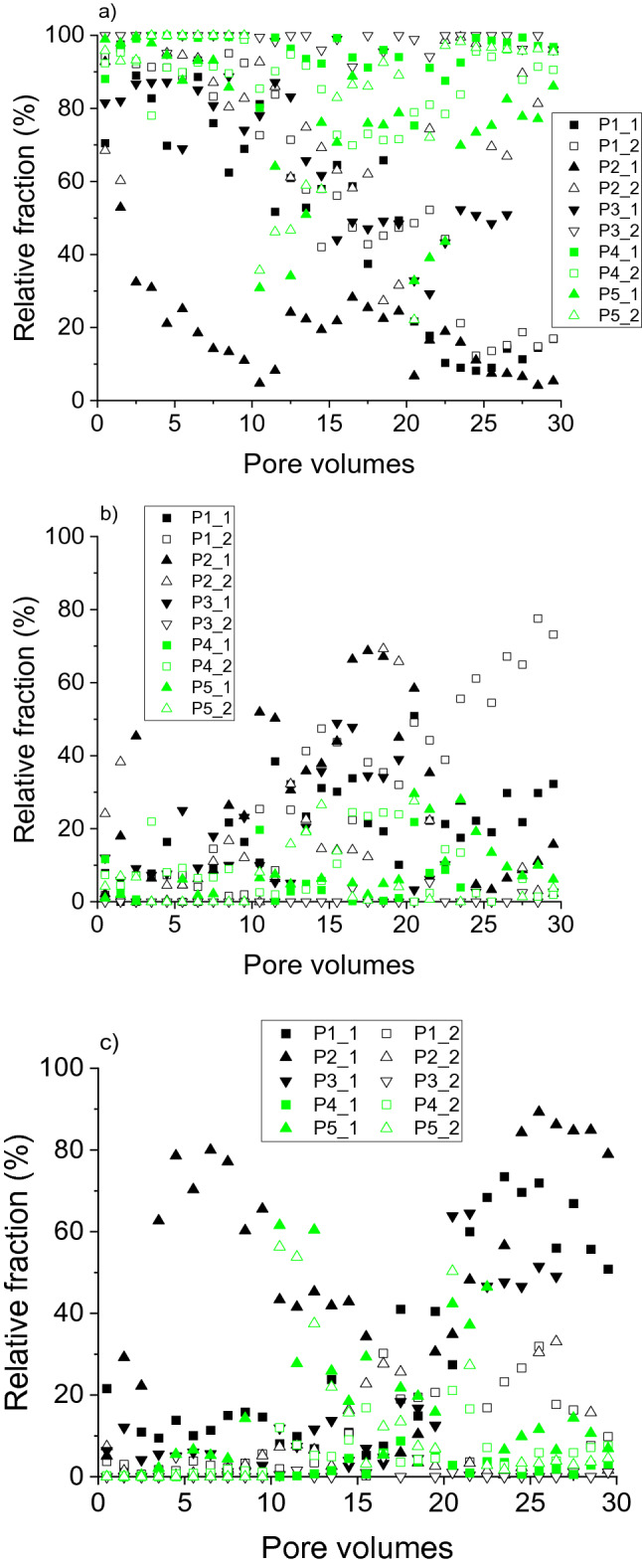
Relative distribution of silicon eluted from soils with increasing degrees of podzolisation (P1 to P5) in irrigation experiments between fractions. (**a**) < 1 kDa, (**b**) 1 kDa–0.45 µm, (**c**) > 0.45 µm. ‘_1’ and ‘_2’ denote duplicate experiments.

The distribution of Si between the size fractions (Fig. 5) differed from that of Al and Fe. Here, apart from effects of the irrigation interruptions, Si < 1 kDa dominated for P4 and P5 (Fig. 5a), with the most advanced degree of podzolisation, consistent with analyses of extracted soil solutions from Podzol E horizons^37^. Decoupled release of Al and Si, with Al predominantly in the 1 kDa– < 0.45 µm fraction and Si predominantly < 1 kDa, excludes transport of SROAS particles to large extent for P4 and P5. On the other hand, particulate Si release may be quantitatively important (> 40% of total Si; Fig. 5c) in all irrigation phases, but with varying samples. Particulate Si in Podzol soil solutions includes amorphous silica and mineral-organic associations, that is, mineral crystallites embedded in an organic matrix like SiO_2_ particles with an approximate size of 100 nm^39^. Si-containing particles transported with the soil solution of Podzols may also comprise phytoliths, which may be as small as 100 nm in plants^34^, and Si-allophanes (molar Al:Si ratio < 0.7)^8^. After the first irrigation interruption, the contribution of Si in the fraction 1 kDa– < 0.45 µm to total Si was important, particularly with P1 to P3. Thus, Si release in the experiments was characterised by a variety of Si species, including monomeric silicic acid (Si < 1 kDa), Si colloids including polymeric silicic acid (1 kDa– < 0.45 µm), and possibly amorphous silica, but there were no clear release patterns for the soils with varying degrees of podzolisation.

**Figure 6:**
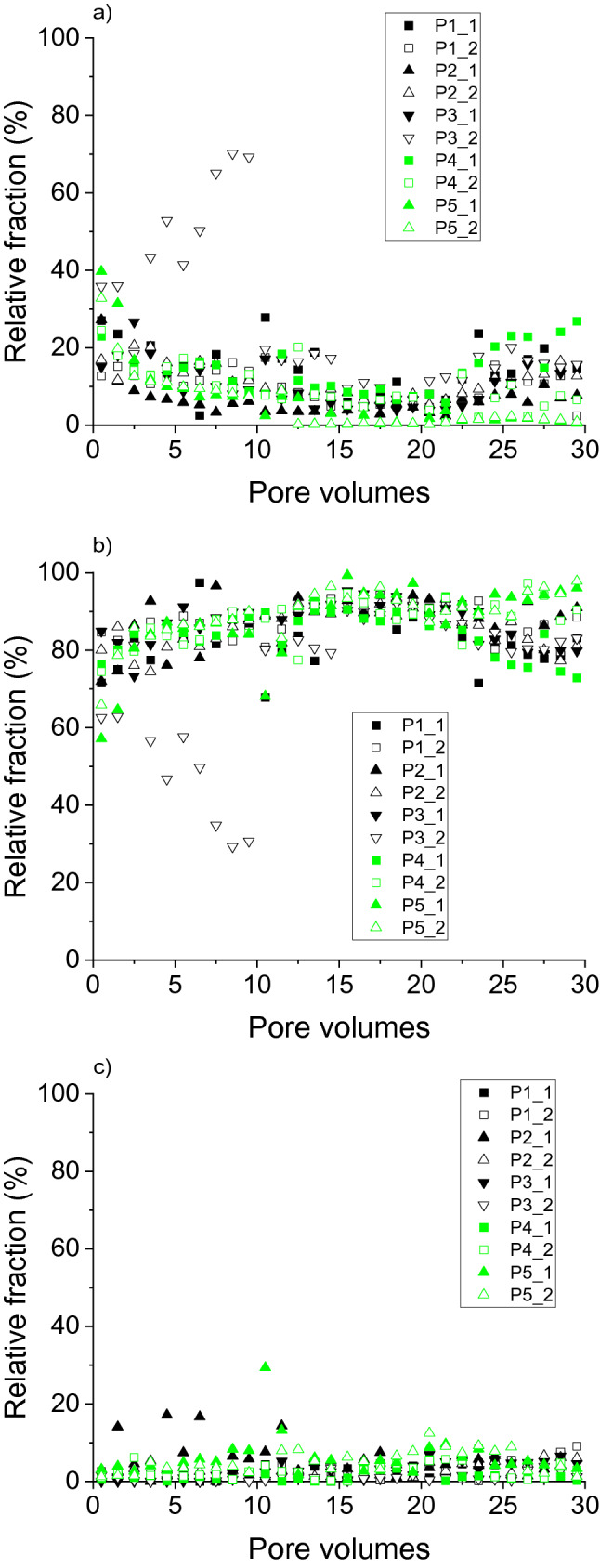
Relative distribution of dissolved organic carbon eluted from soils with increasing degrees of podzolisation (P1 to P5) in irrigation experiments between fractions. (**a**) < 1 kDa, (**b**) 1 kDa–0.45 µm, (**c**) > 0.45 µm. ‘_1’ and ‘_2’ denote duplicate experiments.

Apart from experiment P3_2, the 1 kDa– < 0.45 µm fraction dominated the DOC distribution during the entire experiments with all soils (Fig. 6b). The fraction < 1 kDa was quantitatively more important at the beginning of the experiments and in the final experimental phase (Fig. 6a), and was mostly < 20% of total DOC released, consistent with a previous study^37^. The fraction of particulate DOM > 0.45 µm seldom exceeded 10% (Fig. 6c). However, particulate transport of SOM may be relevant for subsoil SOM accumulation in the long term. Although the majority of SOM in illuvial Podzol horizons was associated with minerals, particulate SOM in illuvial horizons of the soils under study had increasingly interacted with metals with increasing degree of podzolisation, which may have caused flocculation and thus accumulation of the particles^22^. As obvious from the metal:DOC ratios discussed before, DOM release was coupled with that of Al and Fe in the 1 kDa– < 0.45 µm fraction. This fraction contains complexes of metals with larger organic molecules, in addition to poorly soluble aliphatic colloids^30^.

### Are Al and Si transported as SROAS sols from topsoil horizons with varying degrees of podzolisation?

As discussed in both, reviews on Podzol formation and properties and original studies^3,5,17,19,21,40,41^, formation of proto-imogolite or similar SROAS sols as a carrier of Al and Si in vertical elemental transport during podzolisation was suggested. Accordingly, colloidal Si (> 3 kDa) was the dominant form in soil solutions obtained by centrifugation from Podzol E horizons, compared to other horizons^37^. Thus, the association of Al and Si in colloids was suggested in E horizons, but equilibrium calculations excluded SROAS sols as the dominant Al species. Correlation calculations rather indicated Al associated with colloidal SOM^37^, which is consistent with our results for the 1 kDa– < 0.45 µm fraction as pointed out before. Based on analytical fractionation, Al < 0.45 µm in soil solutions of an AE horizon was dominated by organic complexes, and correlation with c(Si) might have indicated presence of SROAS sols^41^.

Preconditions of SROAS formation are sufficient availabilities of aqueous Al and Si. The availability of Al increases at pH < 5 and > 7; particularly pH > 7, as observed for alkaline volcanic ash, provides Al for SROAS formation^34,42^. A Si concentration of the fraction < 1 kDa of 100 µM is the limit for SROAS-sol formation favoured to gibbsite^40,43^. Our data scatter below and above this limit (Supplementary Fig. S3a). In addition, SROAS sols have been suggested even at higher Si concentrations^19^. Thus, the presence of SROAS in our experiments may not be excluded, as the preconditions regarding c(Si) and pH (always < 5; Fig. 2e) were fulfilled.

**Figure 7:**
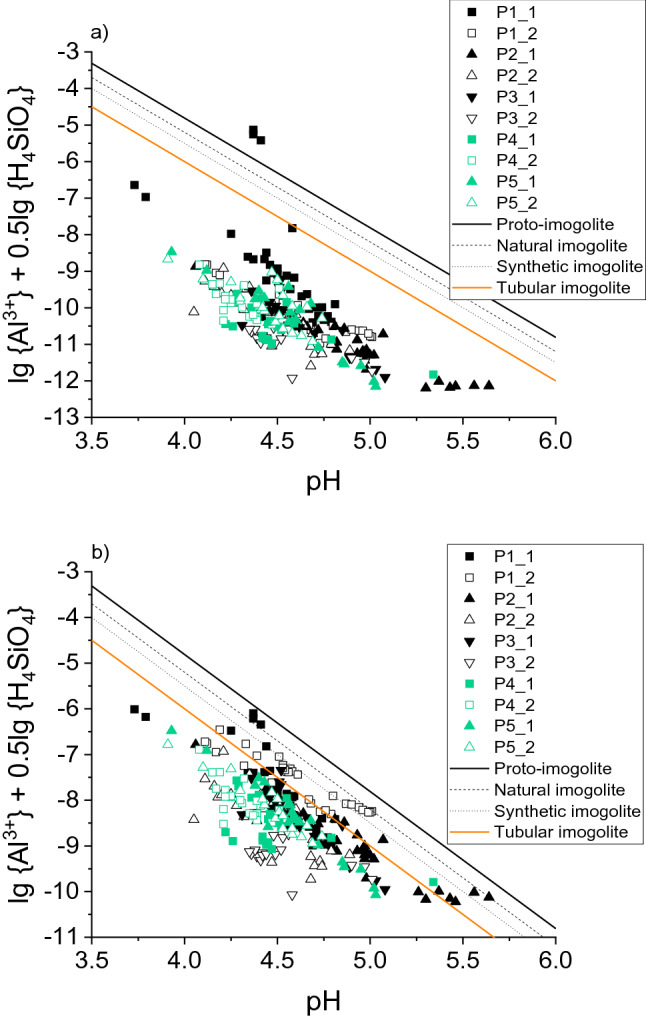
Activities of dissolved Al^3+^ and H_4_SiO_4_ plotted against pH, calculated with (**a**) citric or (**b**) acetic acid as a model of DOM. Lines result from the solubility equation for different types of imogolite^40^, lg {Al^3+^} + 0.5lg {H_4_SiO_4_} + 3pH = lg *K_so_. Logarithmic equilibrium constants *K_so_ (at 298 K) were 7.19 for proto-imogolite^40^, 6.8 for natural imogolite, 6.5 for synthetic imogolite^44^, and 6.0 for tubular imogolite^45^.

Molar Al:Si ratios of SROAS sols were suggested to be approximately two^20^ or ranging from 2.3 to 3.5^40^. In our experiments, the Al:Si ratios of the fractions < 1 kDa and 1 kDa– < 0.45 µm varied over a wide range. Remarkably, the Al:Si ratios of the < 1 kDa fraction never reached the ratios required for the formation of SROAS sols, as they were < 1.3 (P1), < 0.3 (P2, P3), and < 0.2 (P4, P5). As the use of total Al and Si concentrations may not be appropriate for an estimation of the possibility of SROAS formation, we conducted speciation calculations. Using the activities of free Al^3+^ ions and H_4_SiO_4_ in the fractions < 1 kDa, and considering complexation of Al with citric acid (Fig. 7a) as a model of LWOCs, all data points indicate strong undersaturation with respect to four examples of SROAS, apart from four exceptions (P1_1). However, using acetic acid, which may be present in the soil solution percolating in the A horizon of podzolised soils at dominant concentrations^14,27,28^, as a model of LWOCs, several data points indicated equilibrium with one of the model SROAS (Fig. 7b). This was particularly the case with data from experiments with the least podzolised soils (P1, P2), while the points representing the most podzolised soils (P4, P5) plotted away from the equilibrium lines. X-ray amorphous soil constituents, which may include SROAS, were dissolved in a laboratory podzolisation experiment with the A horizon of P1^12^. According to previous studies^17,19,46,47^, undersaturation with respect to SROAS is a common feature of the soil solution of E horizons of Podzols. Consequently, the activities of Al^3+^ and monosilicic acid < 1 kDa may be controlled by dissolution of SROAS in Ah horizons in an early state of podzolisation, that is, prior to the formation of developed E horizons.

The Al:Si ratios of the 1 kDa– < 0.45 µm fractions were distinctly larger (P1, > 1.2; P2, > 0.7; P3, > 1.5; P4, > 0.6; P5, 2.4), up to very large values (> 200), which, however, were caused by very small Si concentrations close to the detection limit (10 µg L^−1^). Although these molar ratios fit well to the Al:Si ratios reported for SROAS, we are very careful with the interpretation of these ratios as a proof of the actual presence of pure SROAS phases in the 1 kDa– < 0.45 µm fraction, given the very likely association of Al (and Fe) with organic colloids, as described in the preceding section. However, a continuum of Al associated with organic colloids and SROAS-DOM co-precipitated phases in this size fraction may be imaginable, but requires further research.

## Conclusions

There was no statistically significant effect of the degree of podzolisation on the distribution of released elements between size fractions. Nonetheless, effects of the irrigation intensity and permanence on element concentrations became obvious, confirming previous findings on the quantitative seasonality of podzolisation processes in soil. These effects of irrigation affected additionally the size distribution of elements, pointing to an accessory qualitative seasonality of podzolisation, which could be studied in the field in more detail. With all soils, Al and Fe release was coupled with that of DOM in the fraction 1 kDa– < 0.45 µm, indicating predominantly complexation with large organic molecules and colloids, rather than Al and Fe complexed by small LMWOCs. Particulate (> 0.45 µm) DOM release and transport was of minor importance. The mobility of Si was decoupled from that of Al, as Si was mostly present < 1 kDa, that is, most likely as monomeric silicic acid. We cannot exclude the presence of SROAS in the topsoil horizons of the least podzolised soils, which possibly control the Al and Si concentrations < 1 kDa. However, there was no direct evidence of the mobilisation and transport of SROAS sols. In the horizons with the most advanced degree of podzolisation, SROAS were depleted so that Si concentrations were likely controlled by dissolution of minerals resistant to weathering, such as quartz and feldspars, and desorption of monomeric silicic acid from polymeric silicic acid adsorbed on minerals.

## Supplementary Information

Supplementary Information.

## Data Availability

The datasets generated and analysed during the current study available from the corresponding author on reasonable request.
