# Supplementary material for: Distribution of Al, Fe, Si, and DOC between size fractions mobilised from topsoil horizons with progressing degree of podzolisation
Source: Sci Rep. 2022 May 19;12:8384. doi: 10.1038/s41598-022-12616-4 (PMC9120154; doi:10.1038/s41598-022-12616-4)
Supplement: Supplementary file 1 — Supplementary Information. [file 41598_2022_12616_MOESM1_ESM.pdf]

Distribution of Al, Fe, Si, and DOC between size fractions mobilised from topsoil horizons with progressing degree of podzolisation

Agnes Krettek, Mathias Stein, Thilo Rennert*

Department of Soil Chemistry, Institute of Soil Science and Land Evaluation, University of Hohenheim, D-70593 Stuttgart, Germany

* Corresponding author, e-mail: t.rennert@uni-hohenheim.de

Figure legends

Figure S1. Aluminium concentrations in eluate fractions from irrigation experiments with soils with increasing degrees of podzolisation (P1 to P5). '_1' and '_2' denote duplicate experiments. a) <1 kDa, b) 1 kDa – 0.45 µm, c) >0.45 µm.

Figure S2. Iron concentrations in eluate fractions from irrigation experiments with soils with increasing degrees of podzolisation (P1 to P5). '_1' and '_2' denote duplicate experiments. a) <1 kDa, b) 1 kDa – 0.45 µm, c) >0.45 µm.

Figure S3. Silicon concentrations in eluate fractions from irrigation experiments with soils with increasing degrees of podzolisation (P1 to P5). '_1' and '_2' denote duplicate experiments. a) <1 kDa, b) 1 kDa – 0.45 µm, c) >0.45 µm. The line at c(Si) = 2.8 mg L^{-1} represents the concentration limit (100 µM) at which formation of SRAOS sols is favoured to gibbsite.

Figure S4. Dissolved organic carbon concentrations in eluate fractions from irrigation experiments with soils with increasing degrees of podzolisation (P1 to P5). '_1' and '_2' denote duplicate experiments. a) <1 kDa, b) 1 kDa – 0.45 µm, c) >0.45 µm.

Figure S5. Squared Pearson correlation coefficients (R^2) for the linear correlation between metal and DOC concentrations in the fractions <1 kDa, 1 kDa – 0.45 µm, and >0.45 µm. a) Al and DOC, b) Fe and DOC.

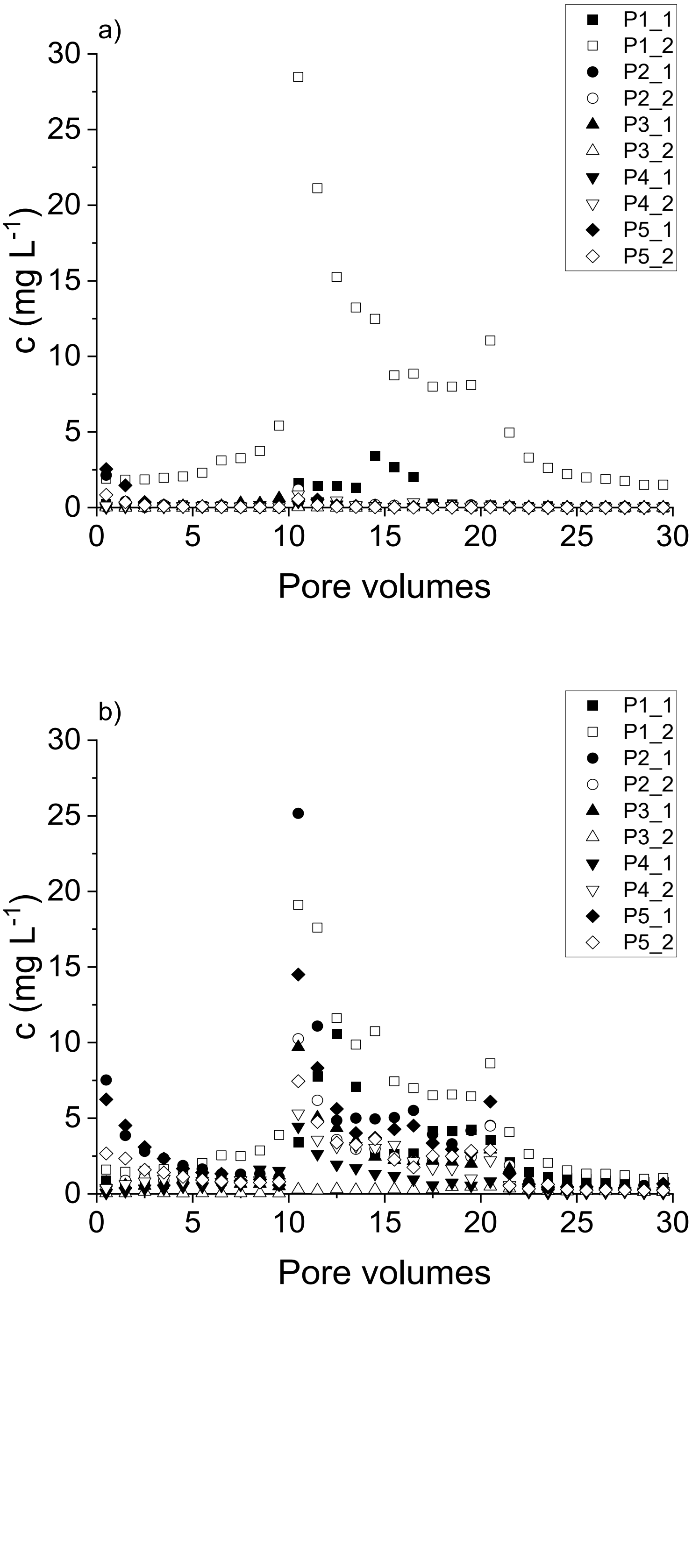
a)
P1_1
P1_2
P2_1
P2_2
P3_1
P3_2
P4_1
P4_2
P5_1
P5_2
c (mg L-1)
Pore volumes
0
5
10
15
20
25
30
b)
P1_1
P1_2
P2_1
P2_2
P3_1
P3_2
P4_1
P4_2
P5_1
P5_2
c (mg L-1)
Pore volumes

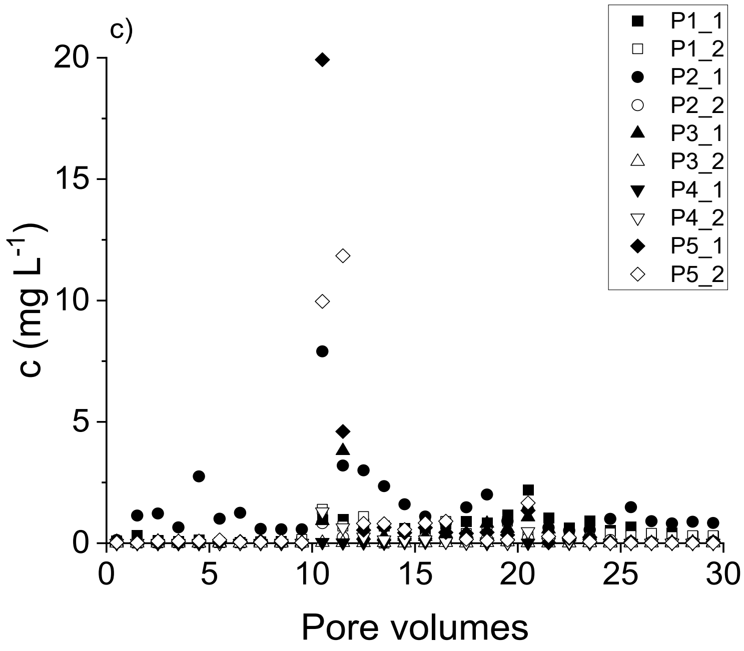

Figure S1. Aluminium concentrations in eluate fractions from irrigation experiments with soils with increasing degrees of podzolisation (P1 to P5). a) <1 kDa, b) 1 kDa – 0.45 µm, c) >0.45 µm. '_1' and '_2' denote duplicate experiments.

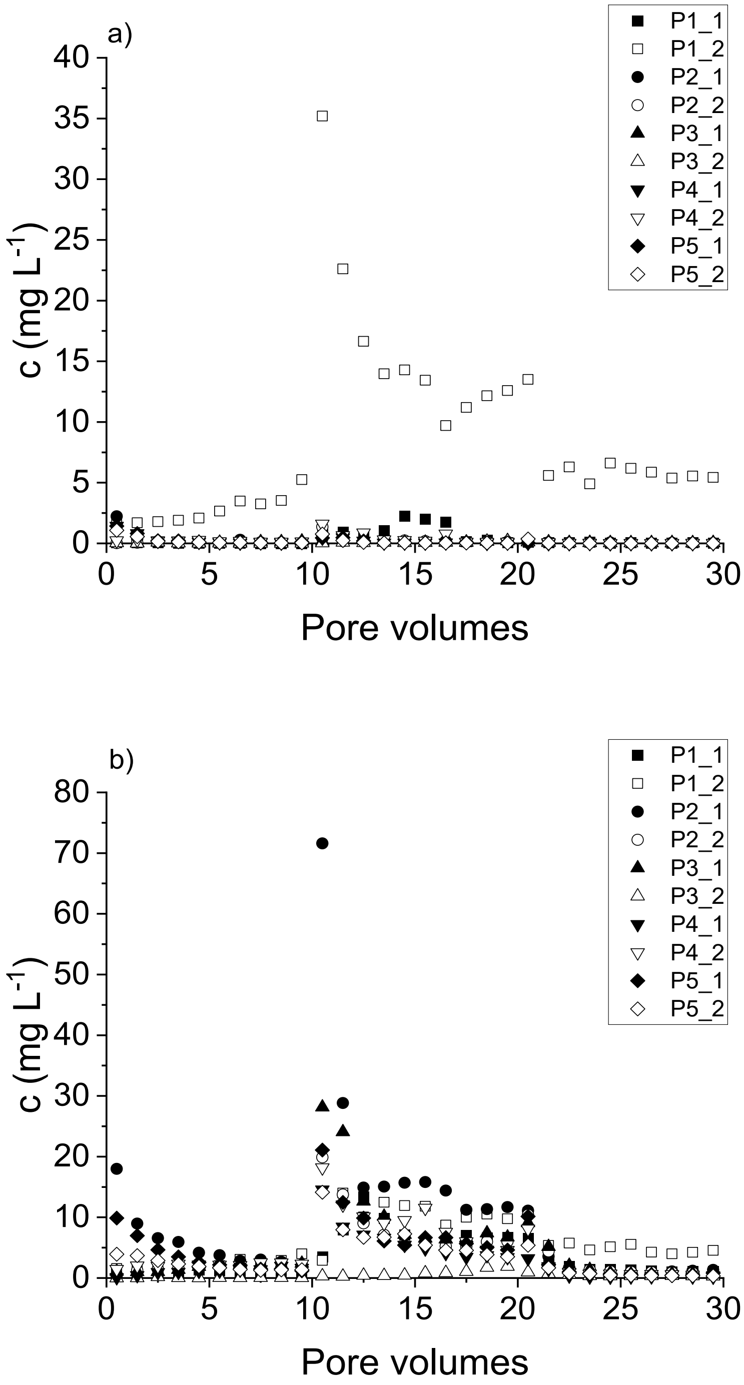
a)
c (mg L^{-1})
Pore volumes
P1_1
P1_2
P2_1
P2_2
P3_1
P3_2
P4_1
P4_2
P5_1
P5_2
b)
c (mg L^{-1})
Pore volumes
P1_1
P1_2
P2_1
P2_2
P3_1
P3_2
P4_1
P4_2
P5_1
P5_2

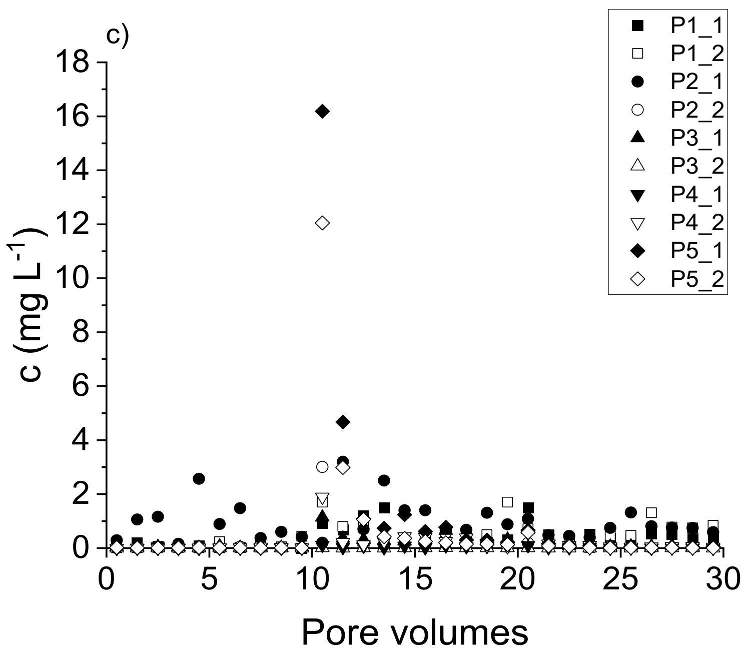

Figure S2. Iron concentrations in eluate fractions from irrigation experiments with soils with increasing degrees of podzolisation (P1 to P5). a) <1 kDa, b) 1 kDa – 0.45 µm, c) >0.45 µm. '_1' and '_2' denote duplicate experiments.

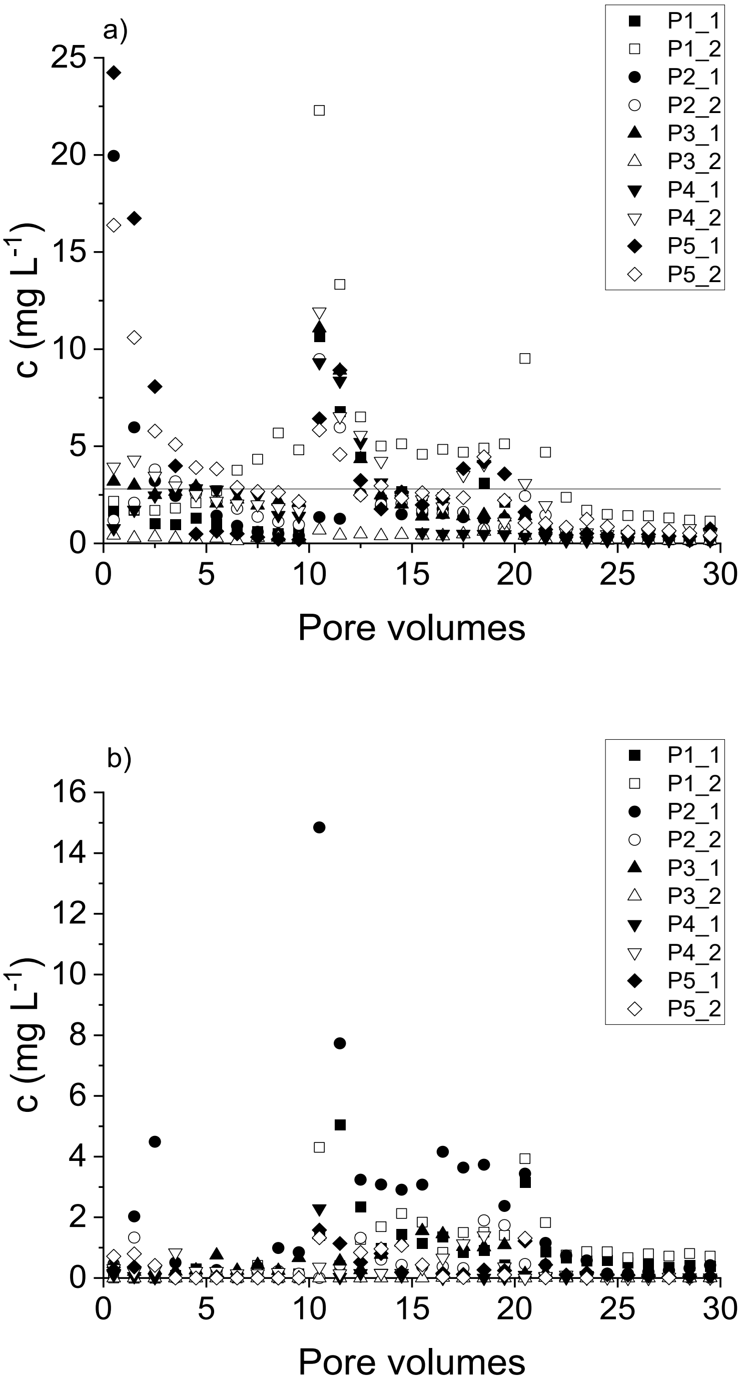

a)
c (mg L-1)
Pore volumes
P1_1
P1_2
P2_1
P2_2
P3_1
P3_2
P4_1
P4_2
P5_1
P5_2
b)
c (mg L-1)
Pore volumes
P1_1
P1_2
P2_1
P2_2
P3_1
P3_2
P4_1
P4_2
P5_1
P5_2

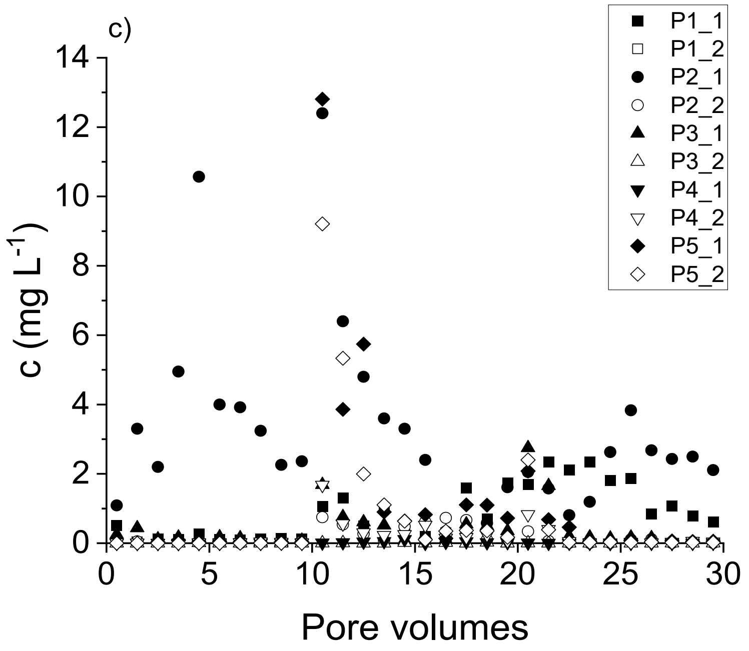

Figure S3. Silicon concentrations in eluate fractions from irrigation experiments with soils with increasing degrees of podzolisation (P1 to P5). '_1' and '_2' denote duplicate experiments. a) <1 kDa, b) 1 kDa – 0.45 µm, c) >0.45 µm. The line at c(Si) = 2.8 mg L^{-1} represents the concentration limit (100 µM) at which formation of SRAOS sols is favoured to gibbsite.

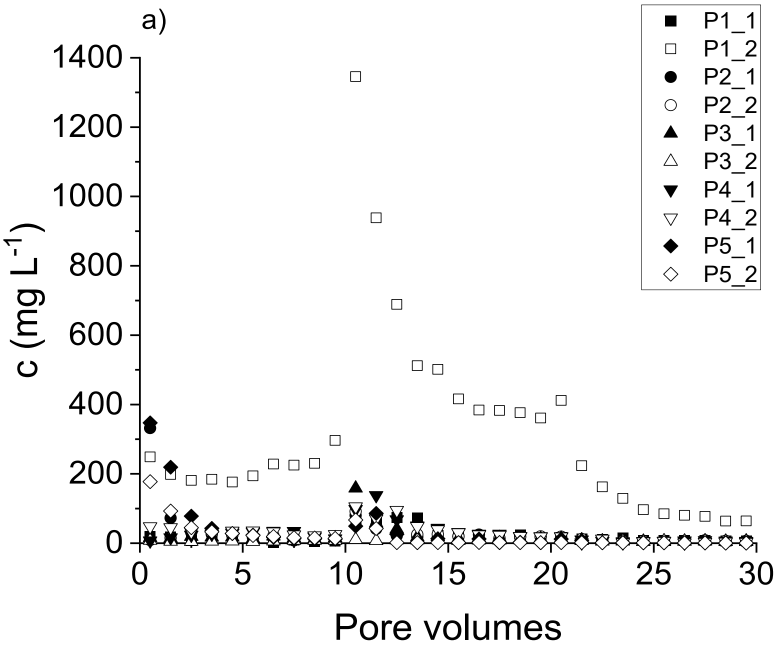

a)
c (mg L-1)
Pore volumes
P1_1
P1_2
P2_1
P2_2
P3_1
P3_2
P4_1
P4_2
P5_1
P5_2

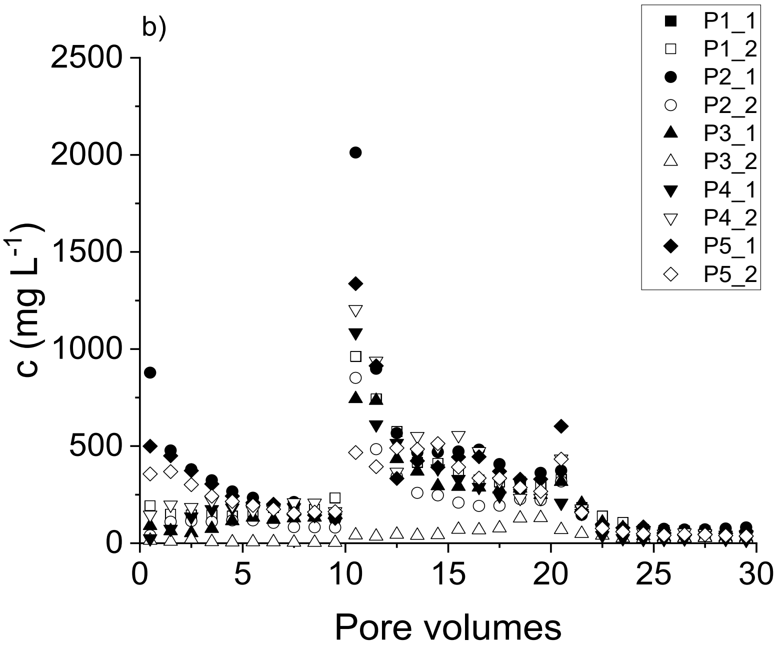

b)
c (mg L-1)
Pore volumes
P1_1
P1_2
P2_1
P2_2
P3_1
P3_2
P4_1
P4_2
P5_1
P5_2

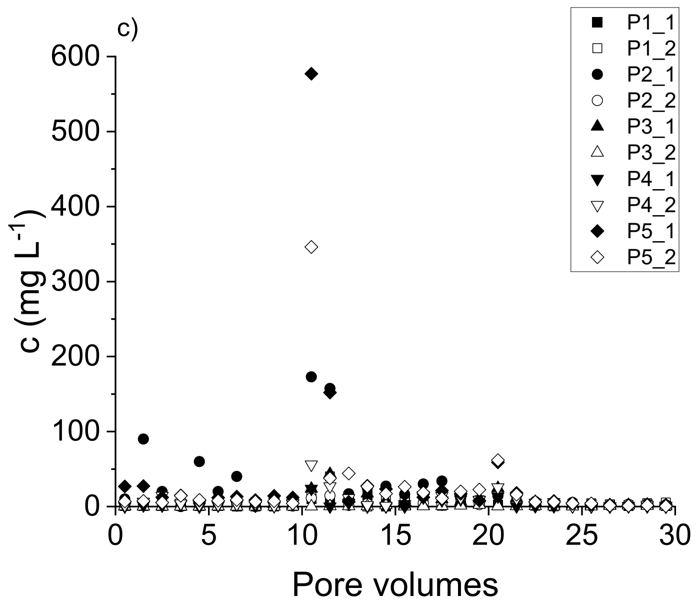

Figure S4. Dissolved organic carbon concentrations in eluate fractions from irrigation experiments with soils with increasing degrees of podzolisation (P1 to P5). a) <1 kDa, b) 1 kDa – 0.45 µm, c) >0.45 µm. ‘_1’ and ‘_2’ denote duplicate experiments.

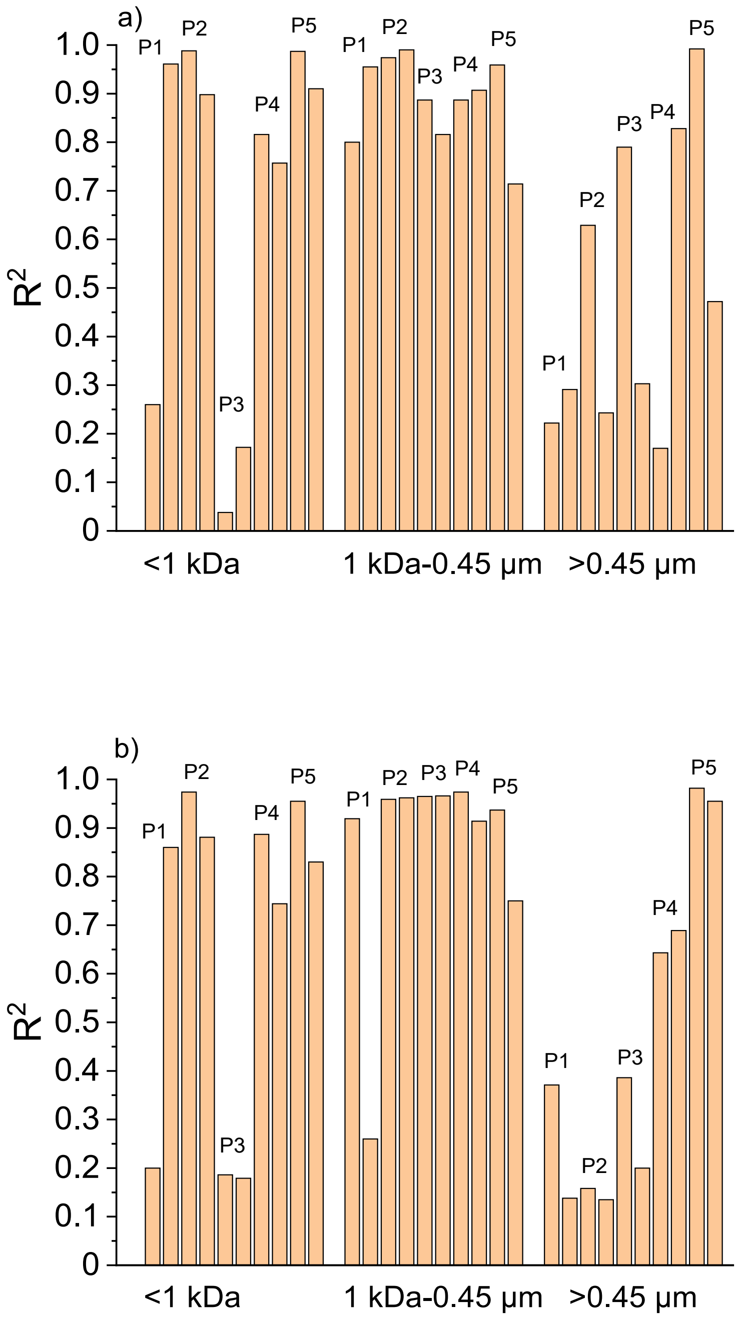

Figure S5. Squared Pearson correlation coefficients (R^2) for the linear correlation between metal and DOC concentrations in the fractions <1 kDa, 1 kDa – 0.45 μm, and >0.45 μm. a) Al, b) Fe.
